# Power of mzRAPP-Based Performance Assessments in MS1-Based Nontargeted Feature Detection

**DOI:** 10.1021/acs.analchem.1c05270

**Published:** 2022-06-07

**Authors:** Yasin El Abiead, Maximilian Milford, Harald Schoeny, Mate Rusz, Reza M. Salek, Gunda Koellensperger

**Affiliations:** † Department of Analytical Chemistry, University of Vienna, Vienna 1090, Austria; ‡ Department of Inorganic Chemistry, University of Vienna, Vienna 1090, Austria; § International Agency for Research on Cancer, Section of Nutrition and Metabolism, Lyon 96008, France

## Abstract

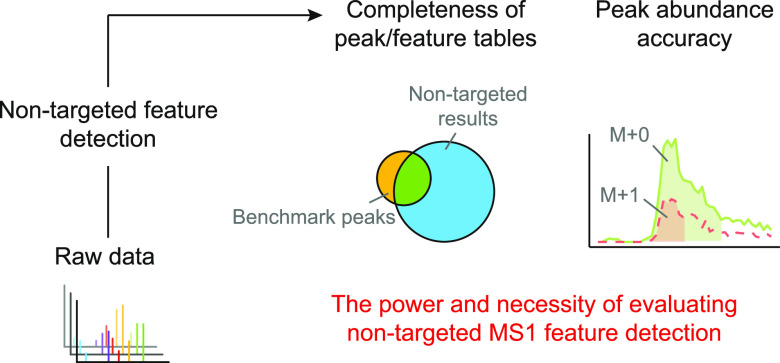

When performing chromatography-mass spectrometry-based nontargeted metabolomics, or exposomics, one of the key steps in the analysis is to obtain MS1-based feature tables. Inapt parameter settings in feature detection will result in missing or wrong quantitative values and might ultimately lead to downstream incorrect biological interpretations. However, until recently, no strategies to assess the completeness and abundance accuracy of feature tables were available. Here, we show that mzRAPP enables the generation of benchmark peak lists by using an internal set of known molecules in the analyzed data set. Using the benchmark, the completeness and abundance accuracy of feature tables can be assessed in an automated pipeline. We demonstrate that our approach adds to other commonly applied quality assurance methods such as manual or automatized parameter optimization techniques or removal of false-positive signals. Moreover, we show that as few as 10 benchmark molecules can already allow for representative performance metrics to further improve quantitative biological understanding.

The exhaustive translation of all chemical ions analyzed via liquid chromatography–high-resolution mass spectrometry (LC–HRMS) into features with accurate MS1-based peak areas precedes any comprehensive data analysis (Figure S1a). Validation tools allowing to assess the completeness and peak abundance accuracy of feature tables are required irrespective of the feature finding tool used (e.g., XCMS,^1^ XCMS-online,^2^ MZmine 2,^3^ MS-DIAL,^4^ El-MAVEN,^5^ OpenMS,^6^ etc.).

Indeed, numerous studies described problems in MS1-based feature tables generated via nontargeted data analysis. For instance, a reanalysis of 5 already published feature tables revealed that each of them omitted >50 relevant compounds due to incomplete feature extraction.^7^ Other studies reported as little as a 10% overlap between feature tables extracted from the same data set when using different tools^8^ or difficulties in reproducing feature tables across different labs.^9^ In this study we further emphasize this problem by demonstrating how marginal differences in XCMS parameter settings can make the difference between missing ∼6% or ∼93% of all peaks in a data set. Overall, the emerging unease regarding the underutilization of data led to several voices calling for solutions, enabling the benchmarking of different tools, algorithms, and parameter choices.^10−12^

Various studies have been published scrutinizing the completeness of MS1 feature tables and the accuracies of the peak abundances reported.^11,13,14^ Generally, these studies are done by defining a ground truth of manually confirmed peaks with known peak abundance ratios, commonly referred to as the benchmark (BM). The errors in the feature tables are then judged by examining the differences between the BM and the feature table. Thereby, in principle, this method allows detecting recovered/missed BM peaks and the accuracy of peak abundances. While the concept is straightforward, it is rarely applied in routine nontargeted experiments, as its implementation can be tedious. This is because BM generation requires meticulous manual curation of peaks, which can be very time-consuming and is often considered to be too subjective for a ground-truth generation. Indeed, a recent study showed that three experts in mass spectrometry strongly disagreed on what constitutes an actual chromatographic peak in ∼20% of cases (*n* = 1071), demonstrating how vague boundaries between differences in opinion carry the risk of overinterpreting differences between BMs and nontargeted feature tables.^15^ Moreover, the manual work of BM curation leads to rather small sets of BM peak lists, potentially hampering the representativeness of the BMs for the whole data set.

We recently introduced mzRAPP, a tool enabling the semiautomated generation of reliable BM peak lists and their fully automated utilization for assessing the completeness (proportion of BM peaks also found/recovered via nontargeted feature extraction; Figure S1b) and peak abundance accuracy (proportion of accurate isotopologue ratios (IRs) as calculated from nontargeted features; Figure S1c) of feature tables at different stages of the nontargeted feature detection (NFD) pipeline (peak picking, peak alignment, gap-filling, and feature filtering).^16^

Briefly, mzRAPP takes the output of traditional targeted metabolomic data evaluation (molecular formulas with associated retention time boundaries) as input for the generation of a BM, which is then utilized to detect feature table errors, as depicted in Figure 1a. The high quality (HQ) of BMs is ensured by comparing IRs calculated from BM peak areas to those predicted from molecular formulas. Thereby, each BM peak can be confirmed to be within the linear dynamic range of the instrumental setup, and IR can be employed as reliable abundance ratios to assess the accuracy of peak areas (see Figures 1b and S2). Overall, mzRAPP can retrieve performance metrics for completeness and peak abundance accuracy for different stages of the NFD process, allowing to assess performances for general parameter selection in peak picking, peak alignment, gap-filling, and feature filtering.

**Figure 1:**
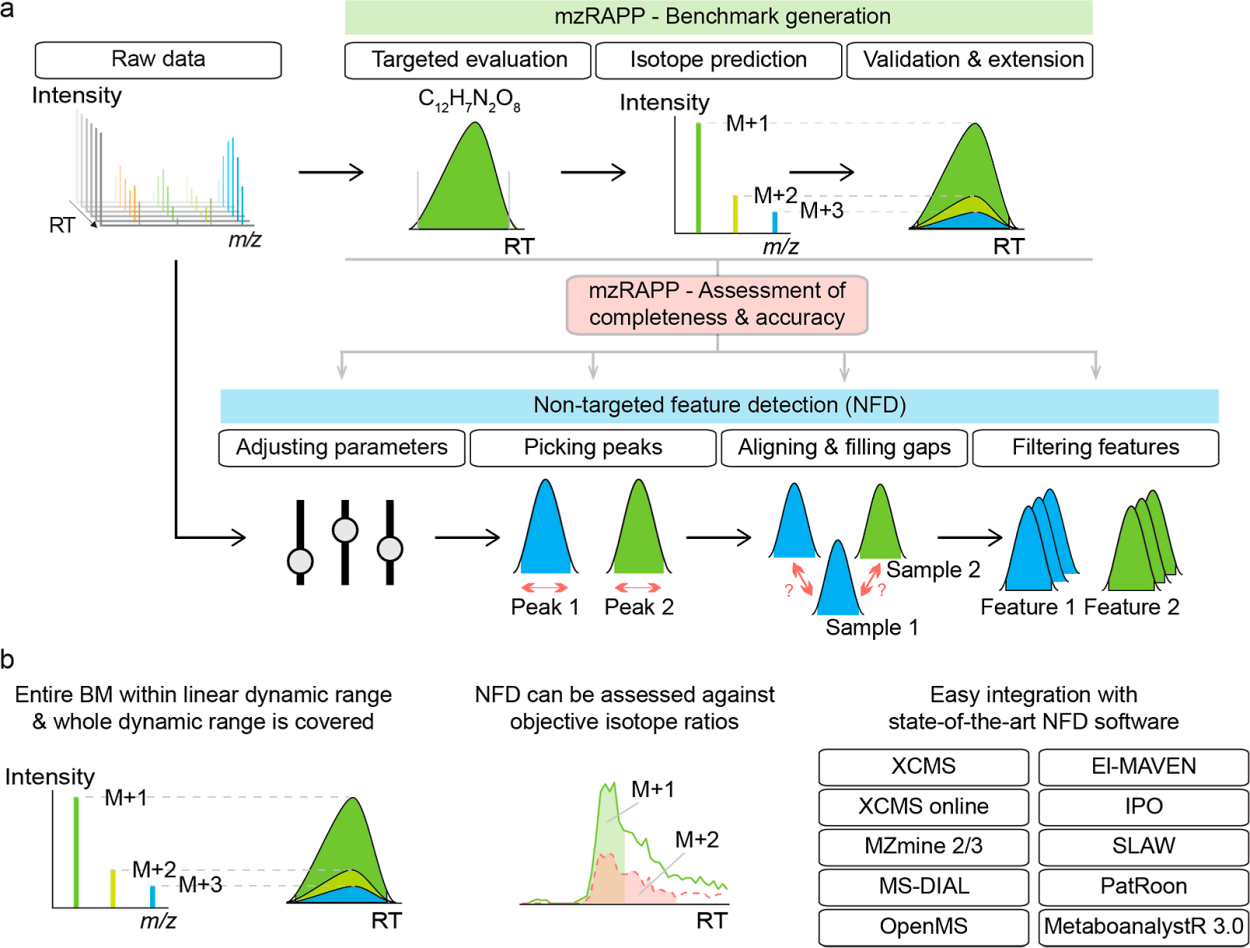
General capabilities of mzRAPP and NFD steps evaluated in the presented study were depicted in (a). Furthermore, key advantages of mzRAPP were highlighted in (b). Briefly, mzRAPPs utilization of isotope patterns allows for an increased dynamic range coverage of BMs while ensuring that all BM peaks satisfy quantitative criteria. Moreover, NFD abundances can be assessed against objective IR instead of subjective considerations (i.e., peak areas as calculated from the shown peaks would not result in an accurate IR). Finally, mzRAPP allows easy integration with many NFD formats.

Figure 1b summarizes mzRAPPs key advantages. Specifically, all BM peaks are ensured to be within the linear dynamic range of the respective instrument. Moreover, mzRAPP utilizes IR as the objective ground truth to assess NFD results and allows a fully automated integration of a range of different output formats by prominent NFD tools. Finally, the most important performance metrics extracted by mzRAPP are the proportion of detected peaks and the proportion of accurate IR. Both metrics are assessed after the peak picking and alignment step, respectively.

In this work, we show the power, necessity, and broad applicability of this novel validation scheme. First, we establish that the mzRAPP BM generation process applies to a wide variety of data sets produced from different sample types and instrumental platforms. Furthermore, we demonstrate that even comparably small BMs produced via mzRAPP allow us to derive reliable NFD-performance metrics. Afterward, we show that expert domain knowledge for parameter optimization or automatized parameter optimization does not guarantee completeness or peak abundance accuracy of feature tables. Finally, we demonstrate that mzRAPPs metrics indeed provide orthogonal information to other feature-table quality assurance strategies such as the utilization of variation in quality control injections or deep-learning facilitated peak shape classification.

## Methods

### Data Sets

Data sets used for BM generation were downloaded from Metabolights^17^ or thankfully provided by the authors of the respective studies.^11,13,15,18−21^ References and/or repository IDs are provided in the column “Reference” of Table S1. Where not already provided as centroided mzML files, raw files were centroided and converted to mzML format via ProteoWizards msConvert^22^ (version 3.0.21045-7732b6429).

### BM Generation

Targeted data evaluation was performed via Skyline^23^ (version 21.1.0.146) for the most abundant isotopologue of each molecule, for which retention time and molecular formula were known in all data sets. Then, manual set retention time boundaries were exported for each molecule and mzML file. These and the mzML files formed the input for mzRAPP, which also extracted other predictable isotopologues for each molecular formula. Only isotopologues with a Pearson correlation coefficient (PCC) > 0.85, below an IR bias (as calculated by peak areas) of 35%, an IR bias (as calculated by peak heights) of 30%, and a difference between ratio bias (height) and ratio bias (area) below 30% points were accepted.

### Extraction of Nontargeted Data Preprocessing Performance Metrics

Extraction of NFD performance metrics was conducted via mzRAPP (version 1.1.6). The exact criteria and rules for matching between signals of the BM and those of the unaligned and aligned NFD outputs can be found in the original mzRAPP publication^16^ and on Github (https://github.com/YasinEl/mzRAPP). IR biases, as calculated from NFD outputs, were considered to be recovered if they were less than 20% points higher than the respective BM bias. Confidence intervals (CIs) (confidence level = 0.99) for all NFD metrics were derived via bootstrapping of BM molecules (*R* = 1000) using boot package (version 1.3-28).

### Application of Nontargeted Data Preprocessing

All NFD experiments were performed via XCMS3 (version 3.14.1) using R 4.1.0 and MZmine 2 (version 2.53). Parameter optimizations were performed manually or via automated optimization tools. The automated optimization tools were IPO^24^ (version 1.18.0), AutoTuner^25^ (version 1.6.0), MetaboanalystR^26^ 3.0, and SLAW^27^ (version 1.0.0). Classification of peaks by quality was performed via NeatMS^28^ version (0.9), which was run via Python 3.7. For the parameter sensitivity study, the coefficient of variance (CV) investigation and the parameter optimization data set (DS) 6 were processed. For the unsupervised clustering investigation, the assessment of NeatMS DS 1 was processed. Additional details are given in the Supporting Information.^29,30^

### Data Analysis and Figures

All further data analysis was performed using R (version 4.1.0) and R studio (version 1.4.1717) using data.table package. Plots were generated using ggplot2, patchwork, and ggradar. Figures and diagrams were further processed using Adobe Illustrator (25.3.1).

## Results and Discussion

### Quality of Automated BM Curation and Extension

In this study, BMs were generated from 12 different public and in-house raw data sets (listed in Table S1) via mzRAPP. The case-by-case generated BMs covered five different MS-systems coupled to hydrophilic interaction chromatography or reversed-phase chromatography and different sample types (including analytical standard mixtures, blood serum, red blood cell extracts, and cell culture extracts) and compound classes (polar metabolites, lipids, and exogenous small molecules). Targeted extraction of the most abundant isotopologue of each known molecule was done manually but was automatically extended to all lower-abundance isotopologues. Quantitative properties of the thereby generated BMs are visualized in Figure S3. In Figure S3a, all 50597 BM peak areas of low abundant isotopologues (LAITs) were plotted against the area predicted from the respective most abundant isotopologue (MAIT), visualizing the concept that only peaks within the linear dynamic range of the instrumental platform were added to the BMs. Figure S3b shows the absolute peak area bias of all LAITs, with 94% of all calculated IR biases <25%. A comparison of biases (Figure S3c) as calculated via peak areas versus peak heights (which are generally more robust as they are not affected by the poor setting of RT boundaries) revealed a good agreement, further strengthening the evidence of an accurate “ground truth” for an extensive number of peaks. Finally, for DS1, the BM reliability was evaluated upon comparison with reported fold changes assessed in an independent laboratory. Figure S3d shows the excellent reproducibility of the mzRAPP approach applied here. For this specific data set, the number of peaks with reliable quantitative properties increased by > 200% by integrating LAITs. The addition of LAITs increased not only the BM size but also the covered dynamic range. Figure S4a,b quantifies this significant extension for all 12 investigated data sets. This shows how even small manual efforts can lead to large BMs. Peak metrics such as the full width half maximum (FWHM) of chromatographic peaks and the mass precision given by *mz* ranges of individual peaks are important for any nontargeted experiment. In fact, most tools enabling nontargeted MS1 feature extraction require parameters corresponding to these variables for any data set to be processed. Therefore, it is worth noting that the generated BMs showed large differences in all these metrics as a result of different measurement methods (see Figure S4c,d). Next to these characteristics, the peak shape, as reflected by the zigzag index,^31^ sharpness,^32^ and other metrics (Figure S5), show significant differences across investigated data sets. This highlights that data sets can vary significantly in their characteristics and might therefore pose different challenges for NFD. Consequently, conclusions drawn for the performance of a given NFD experiment performed on one raw data set might not allow drawing conclusions for other raw data sets.

### Application of BMs for Feature Extraction Assessment

Ultimately, a BM can be utilized to derive performance metrics for NFD performed on the same raw data set. As outlined above, mzRAPP enables automated assessment of the proportion of found BM peaks and the proportion of accurate IR before and after alignment as performance metrics (see Figure S1b,c).

**Figure 2:**
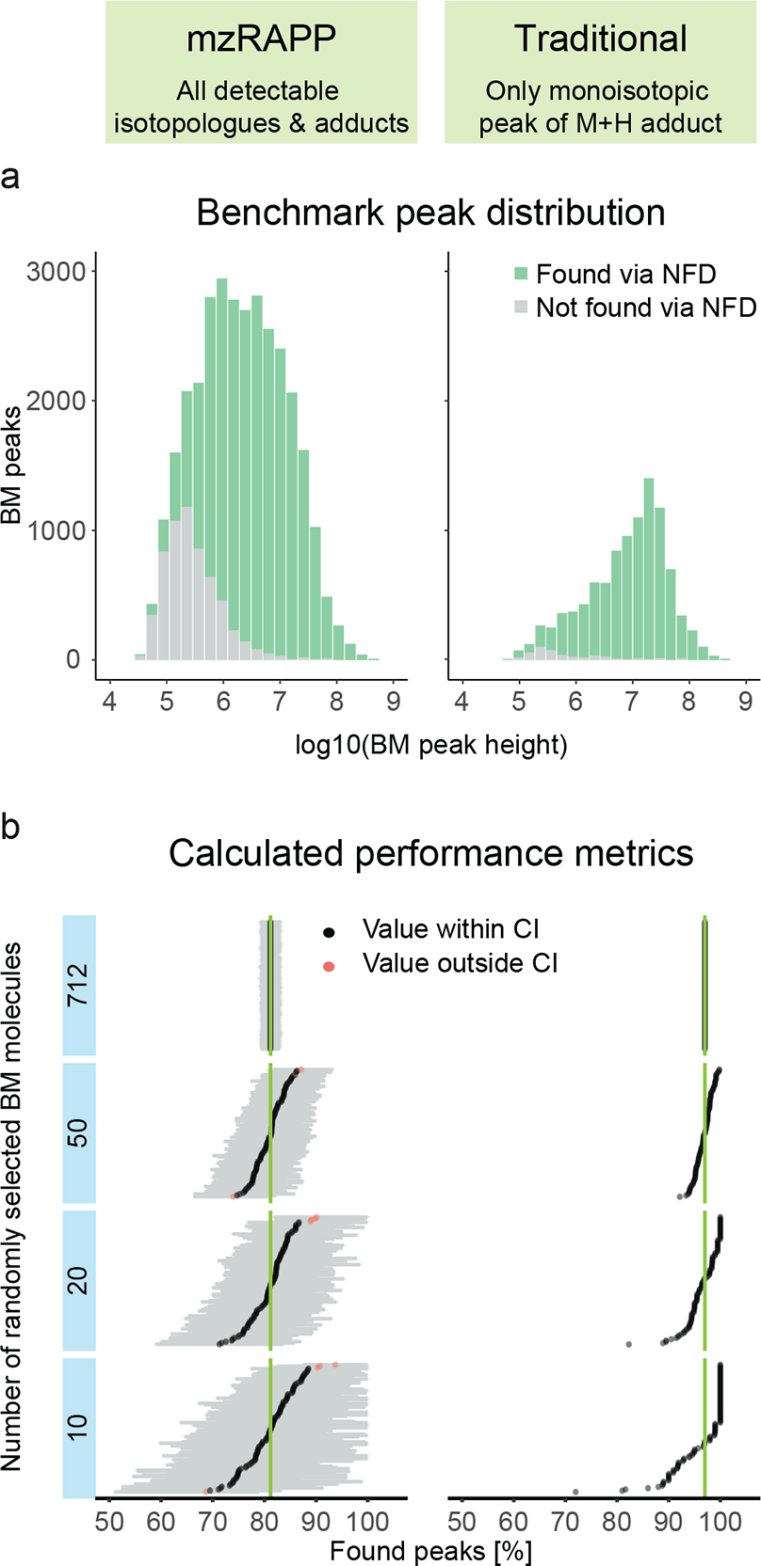
NFD was performed on a raw data set. For the same data set, two BMs were generated from 712 internal known molecules. One BM was generated in a traditional manner, only considering the monoisotopic peak and the M + H adduct, while mzRAPP considered all isotopologues and a range of adducts. Both BMs were used to estimate the proportion of peaks found via NFD. (a) Distribution of peak heights for the two different BMs. (b) Different numbers of molecules were sampled at random from all 712 BM molecules (*n* = 100 for each number of molecules) to estimate the proportion of found peaks after alignment. Only mzRAPP allows the estimation of CIs.

However, metrics derived from the BM (e.g., *x* % of BM peaks found) should be translatable into an estimation for the underlying data set (e.g., *x* % of all peaks found). Hence, a representative sampling of the BM peaks/features is a prerequisite. To show the validity of our approach, we provided an overview showing how mzRAPP compares to more traditional BM recovery studies for the evaluation of an NFD experiment in Figure 2. Figure 2a shows how mzRAPPs consideration of all detectable isotopologues and multiple adducts allows for a better coverage of the linear dynamic range. This is of importance as low peaks are often underrepresented in BMs, only including the MAITs. The potential impact of this underrepresentation is visualized in Figure 2b for different numbers of BM molecules. As can be seen, only considering MAITs leads to a rather consistent overestimation of the proportion of found BM peaks for any number of BM molecules.

Moreover, mzRAPP allows to estimate CIs for all metrics by bootstrapping BM molecules (see also Figure S6). This was done by bootstrapping different numbers of molecules from BM 1 (containing 712 molecules and >30,000 peaks). It can be observed how a reduction of the number of BM molecules increases the CI, while the assessed metric was in agreement with the best value derived from the largest BM (712 molecules) in almost all cases. Therefore, even <50 BM molecules can lead to reasonable estimates of the performance of NFD, as long as the increase in the CI can be accepted.

### Sensitivity of NFD Extraction Parameters

NFD requires the adaptation of different parameters to the analyzed data set. These parameters can appear more or less intuitive to users with different scientific backgrounds and experiences. Generally, parameters involving expected chromatographic peak widths and retention time shifts are often considered to be among the more intuitive parameters. In the following, we showcase examples that emphasize the need for case-by-case benchmarking strategies, as even intuitive parameter settings could have an adverse impact on NFD.

**Figure 3:**
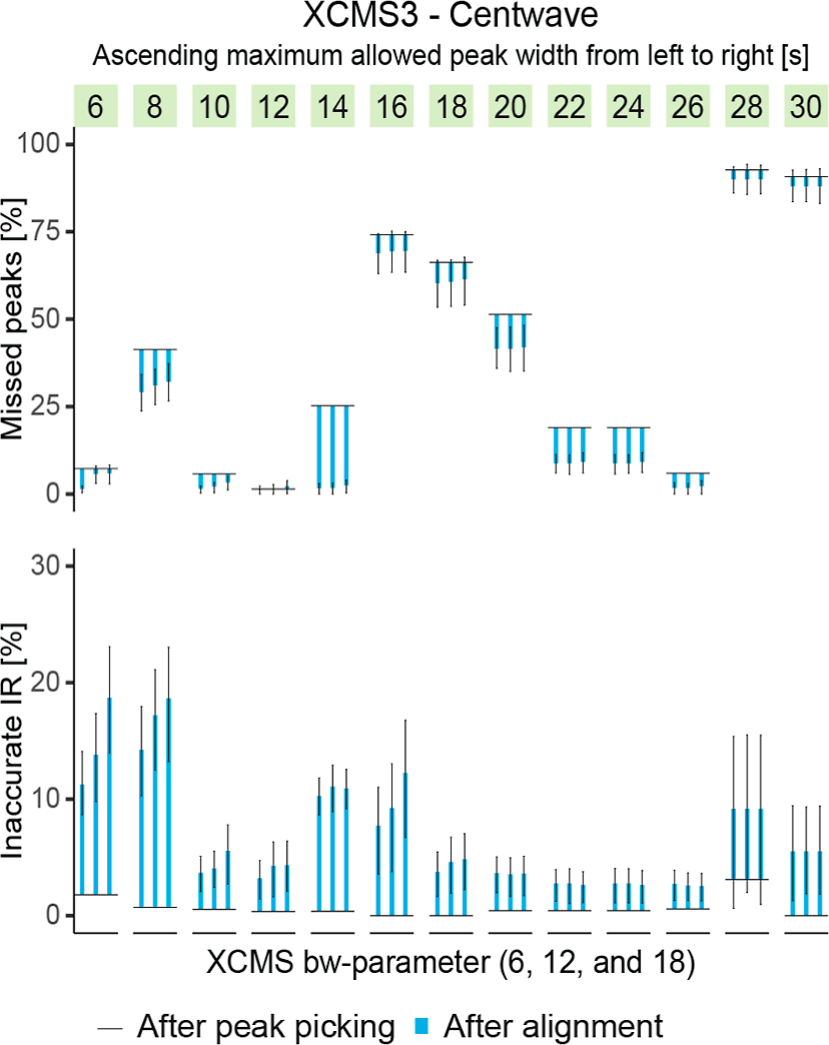
Multiple nontargeted feature detection experiments with parameter sweeps for the peak-picking and the alignment step were performed on the same data set via XCMS3. The maximum peak width (MPW) allowed was incrementally increased, with three retention time tolerances tested for each peak picking attempt. The proportion of recovered BM peaks was strongly and often abruptly affected by the MPW parameter. While the proportion of inaccurate IRs was more strongly influenced after alignment, the effect was primarily dependent on the peak width parameter set for peak picking.

Figure 3 shows how a stepwise increase of XCMS’s centwave’s maximum peak width (MPW) parameter using 2 s increments heavily affected the proportion of missed BM peaks and accurate IR. In the most extreme case, an increase in MPW from 26 to 28 s led to an increase in the proportion of missed BM peaks (before alignment) from 6 to 93%. Considering that the median of BM peaks FWHM ranged from ∼4 to ∼18 s with a median of ∼7 s, there was no trivial dependence of the optimal MPW on the FWHM of peaks to be detected. While fewer peaks were missed after alignment and gap-filling, this improvement was insufficient to make up for errors introduced during peak detection. It is worth noting that even in cases where gap filling recovered most peaks, such as with an MPW of 14 s, the resulting peak areas led to less accurate IR than when peaks were already detected in the peak detection step (e.g., with MPW set to 12 s). While the highest observed retention time shift in the BM peaks was below 10 s, the maximum allowed shift, as set via the bandwidth (bw) parameter in the group density algorithm, did not affect NFD to the same extent as MPW. Interestingly, there was almost no effect of the set MPW on the IR accuracy after peak picking. However, there was a significant impact on IR accuracy after peak alignment and gap-filling, which depended on the MPW set during the peak picking step rather than set alignment parameters.

For this specific dataset, the optimum of all parameter sets tested was found by XCMS (an MPW of 12 s, and a retention time tolerance of 6 s, leading to a proportion of missed BM peaks <1% and a proportion of inaccurate IR < 5%). It should be noted that this finding cannot be generalized, but it holds true for the processed data set, representing a use case of parameter adjustment. An additional case study utilizing MZmine 2 is provided in Figure S7. The example clarifies that the common practice of manual parameter adjustment to metrics derived from the analytical performance of the instrumental setup can lead to suboptimal NFD.

### Application of Parameter Optimization Tools

**Figure 4:**
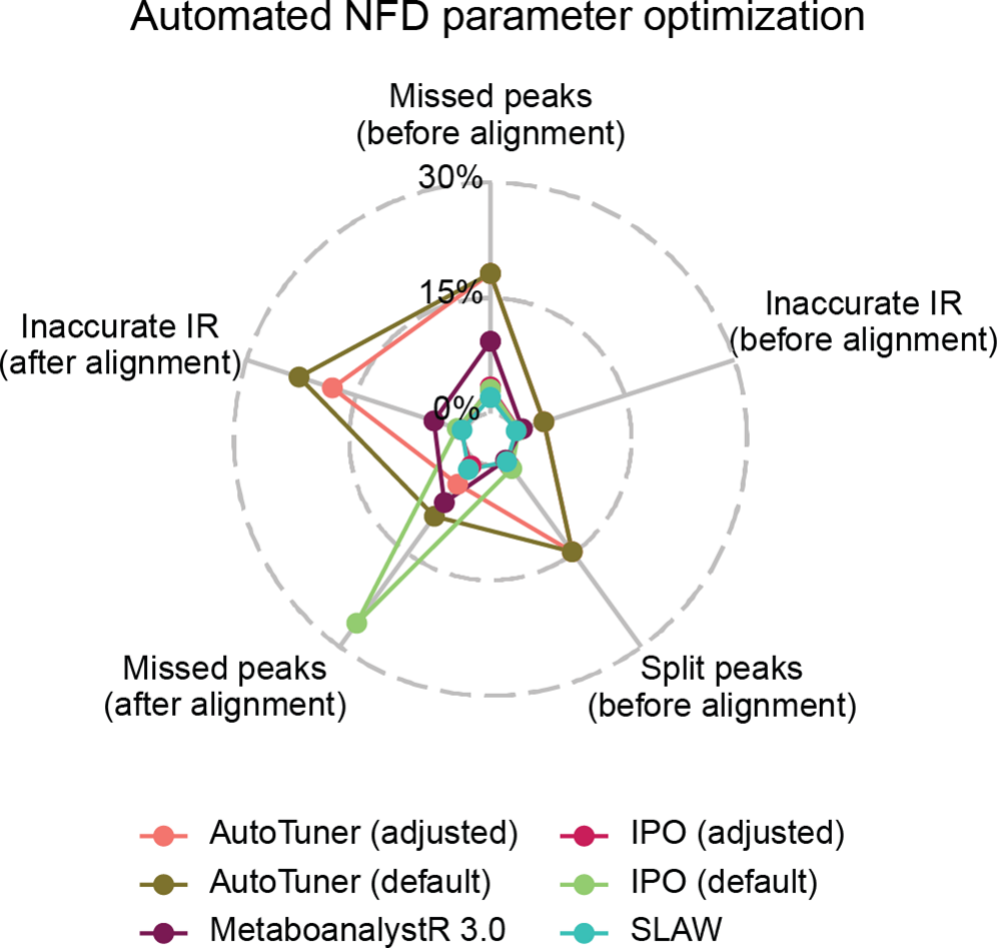
Nontargeted feature detection (NFD) parameters for processing a data set have been optimized using different optimization tools (IPO and AutoTuner, both adjusted and default, as well as MetaboanalystR 3.0 and SLAW). Outcomes were assessed via five BM recovery-based metrics, namely the proportion of missed BM peaks (before and after alignment), the proportion of inaccurate IRs (before and after alignment), and the proportion of split peaks (before alignment). CIs of all metrics for the underlying data set are given in Table S2.

Current parameter optimization algorithms (as implemented in IPO, AutoTuner, MetaboanalystR 3.0, and SLAW) undoubtedly facilitate the NFD extraction and improve quality. Here, we test these tools applying our BM-recovery approach. This way, the otherwise missing metrics of missing peaks and accuracy of peak abundances are validated. Figure 4 compares the quality of parameter optimization performed for the NFD performed on DS 6 via different metrics, as exported by mzRAPP. As can be seen, the differences between the optimization attempts were observable for the proportion of missed BM peaks and inaccurate IR before and after alignment and the proportion of BM peaks leading to split peaks (peaks with borders set close to peak apex). It turned out that the initially defined values for the parameter optimization process are crucial and were unique for each tool. For example, in the case of IPO, manual adjustment of starting parameters led to a decrease in the proportion of BM peaks missing after alignment from ∼25 to ∼1%. Again, this test emphasizes that orthogonal evaluation is not redundant when using automated parameter optimization algorithms.

### Feature Extraction Assessment or Filtering via CV

**Figure 5:**
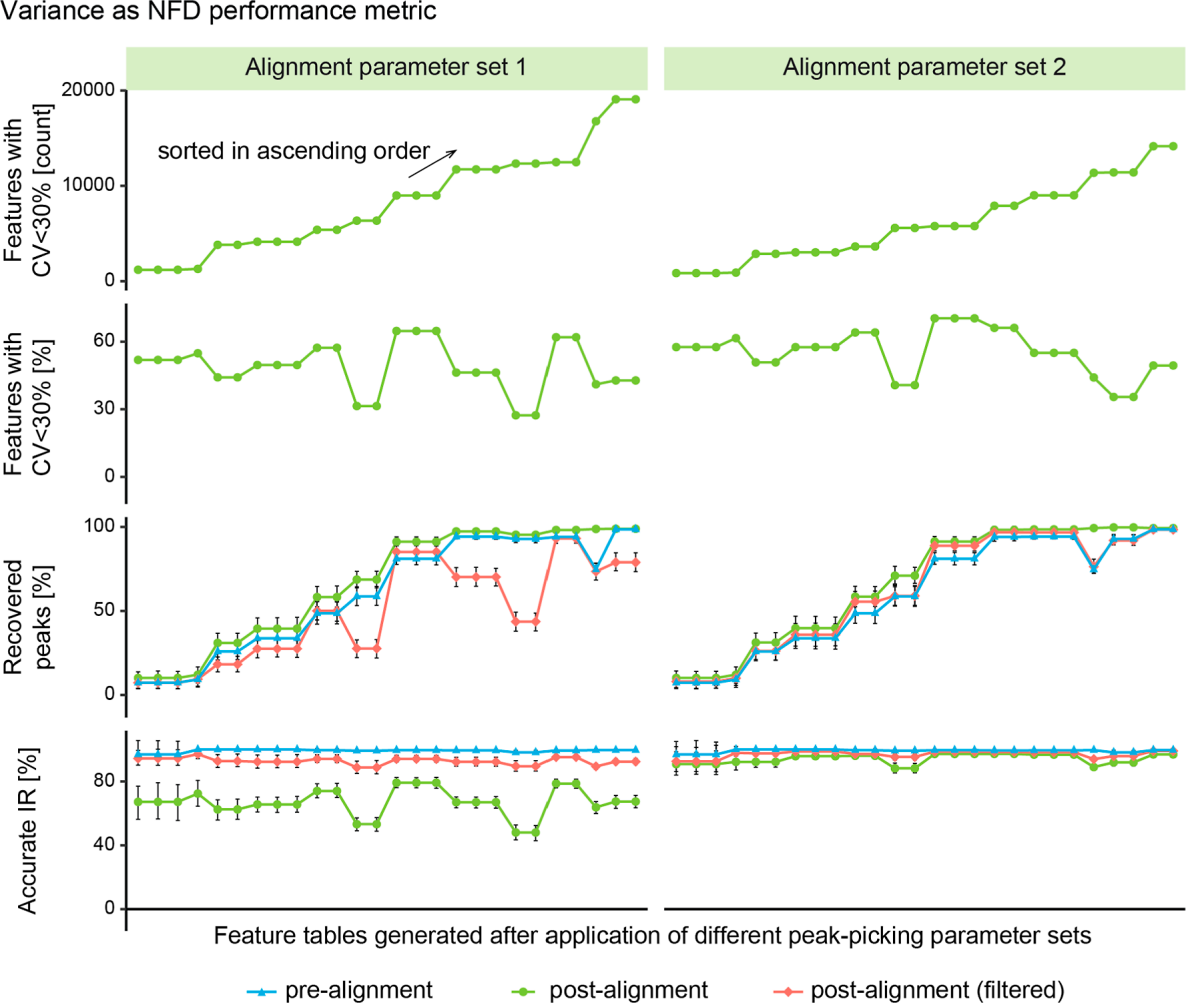
A data set consisting of 9 replicate injections was processed via XCMS using different values for the MPW parameter and the bw parameter, leading to a total of 52 nontargeted feature detection experiments. Four different quality metrics including the number of features with CV < 30%, the proportion of features with CV < 30%, and the proportion of recovered BM peaks and accurate IRs [prealignment, postalignment, and postfiltering (only features without missing values and with CV < 30%)] were then plotted (sorted by ascending number of features with CV < 30%).

Obtaining many features with a low peak abundance variation CV across replicate injections is commonly considered to indicate a good NFD performance. Here, we investigated how CV-based and BM-based NFD performance metrics compare for different NFD parameters. The CV-based metrics considered the number and proportion of features with a CV < 30% (nCV30 and pCV30). mzRAPP metrics addressed completeness and peak abundance accuracy inferred by BM peak recovery and IR accuracy, respectively. In Figure 5, these 4 metrics were plotted for 52 different feature tables generated via NFD using 26 different sets of peak-picking parameters, combined with one of two alignment parameter sets (APSs). Within the two investigated APS, the metric nCV30 was well-correlated with the proportion of BM peaks recovered postalignment (e.g., PCC = 0.9 for APS 1), while the proportion of features with CV < 30% reflected the proportion of accurate IR postalignment (e.g., PCC = 0.97 for APS 1), showing the overall validity of both approaches. However, while CV metrics were very similar between the APS 1 and 2, BM metrics revealed that the proportion of an accurate IR (post alignment) were significantly higher with APS 2. As a key advantage, mzRAPP metrics allow to dissect the single steps of NFD, such as peak-picking (prealignment) and peak alignment (postalignment), while CV-based metrics can only be assessed postalignment. This fact is significant as it allows to derive information on whether the peak-picking or alignment parameters require further optimization. Therefore, comparing the proportion of an accurate IR pre- and postalignment for APS1 reveals that alignment parameters require optimization, which was confirmed as APS2 improving the proportion of an accurate IR significantly. Moreover, BM metrics reveal whether a global optimum has been reached (e.g., 100% of peaks recovered and 100% of IR accurate), while CV metrics only allow to compare the performance across tested parameters. Thus CV metrics do not offer concise decision points for the assessment of a complete and abundant accuracy when optimizing NFD.

However, the removal of features with missing values or a CV > 30% was demonstrated to be a viable filter for removing features with an inaccurate IR while retaining features with an accurate IR (red line). Still, it came at the prize of removing real peaks with poorly set integration boundaries as visible from the drops in the recovered peak metric when many features containing an inaccurate IR had to be removed. Nevertheless, this demonstrates that filtering by the CV of replicate injections was indeed successfully removing unreliable features.

### Application of Peak Classification via Deep Learning

**Figure 6:**
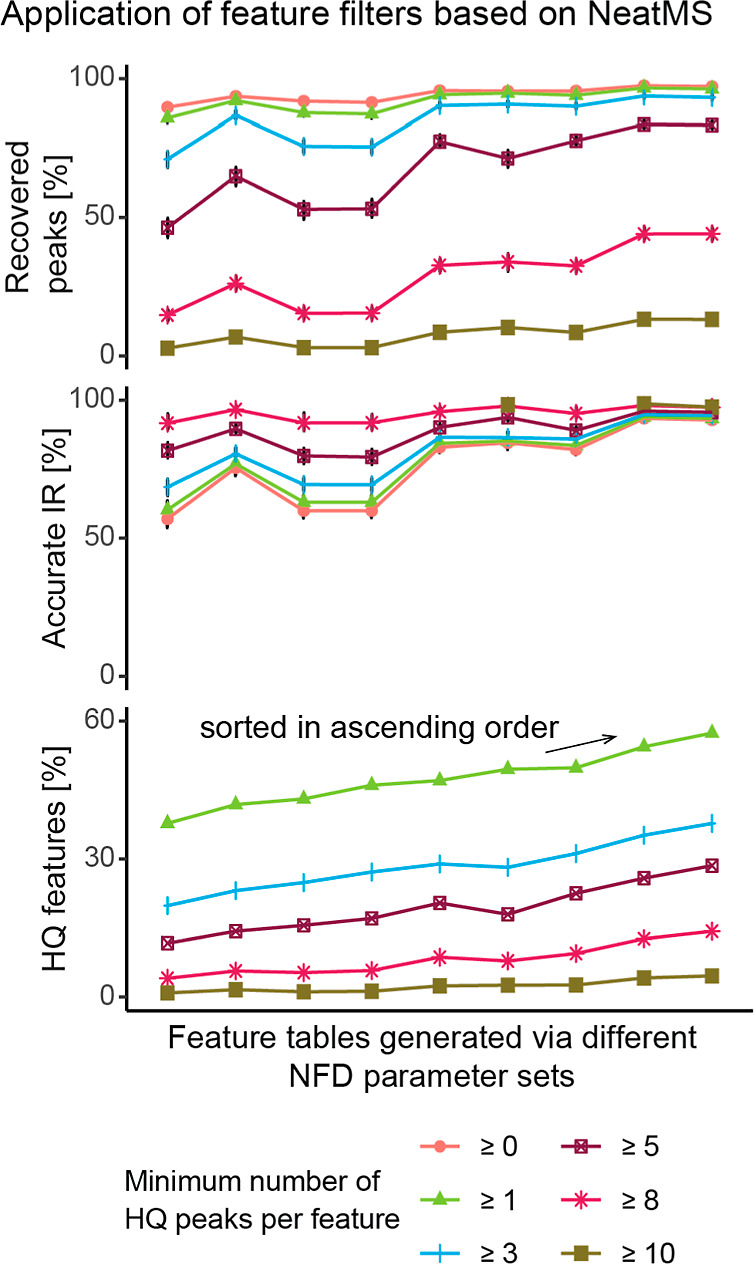
A data set, consisting of 10 samples, was processed with 9 different sets of XCMS-parameters. All peaks produced via XCMS were classified by NeatMS into different categories, including HQ or noise. Different numbers of peaks within an aligned feature were required to be of HQ for a feature to be retained. The plot on the bottom shows the proportion of all XCMS features satisfying these criteria for each parameter set. The plots above show metrics on the proportions of recovered BM peaks and accurate IRs. The *x* axis was sorted by ascending values of HQ features [%] for more than or equal to 1 HQ peak.

Novel tools such as NeatMS use deep learning for the classification of peaks extracted via NFD by their quality. As a major breakthrough, noise removal is accomplished without relying on replicate injections or manual curation. The successful application of deep learning algorithms requires good training data, which (in the case of NeatMS) have to be labeled by users with different skill sets. In this work, we scrutinized NeatMS. Peaks generated via nine NFD experiments performed on the same data set (DS 1; containing 10 samples) were classified accordingly into three categories “high quality,” “low quality,” and “noise.” We then applied different filters to the aligned NFD features and required them to contain 0, 1, 3, 5, 8, or 10 “high quality” peaks. Subsequently, the proportion of recovered peaks and accurate IR after alignment was assessed. For this purpose, we filtered our BM to contain only features with peaks in all 10 samples. As can be seen in Figure 6, removing all NFD features which did not include at least 1 “high quality” peak reduced the number of features by ∼40 to ∼60% while having almost no effect on the proportion of recovered BM peaks or accurate IR, demonstrating how NeatMS can be applied successfully for removing false positives from NFD results. However, requiring more “high quality” peaks reduced the proportion of recovered BM peaks by multiple % points in many cases. When all 10 samples were required to contain only “high quality” peaks for a feature to be retained, the proportion of recovered BM peaks dropped to <10% in all cases. Our validation confirms that tools such as NeatMS for efficiently removing false-positive signals from NFD results have the potential to significantly advance NFD. Despite this undisputed role, the quality and size of training data strongly affect the procedure and are defined by a user, case-by-case. Thus, independent validation such as BM-recovery studies continues to be of great value in any NFD pipeline.

## Conclusions

We conclude that routine performance checks continue to be necessary to ensure the completeness and peak abundance accuracy of feature tables produced via NFD. This conclusion is based on the demonstration that neither manual nor automatized parameter optimization guaranteed optimal outcomes by metrics discussed here and by other studies referenced above. We showed that the BM recovery-based validation as implemented in mzRAPP offers a viable solution to assess the performance of NFD routinely and on a step-by-step basis (e.g., peak-picking, peak alignment, gap-filling, and feature filtering). Finally, we want to emphasize that NFD should indeed be validated on an experiment-by-experiment basis rather than ranking NFD tools by the performance and only applying the ascribed “winner” in the future analysis. This is because the ranking of NFD tools at their peak performance requires unpractical amounts of parameter screening, performances naturally vary across data sets, and NFD tools are updated on a regular basis, making the validity of the performed ranking potentially short. Routine assessments, on the other hand, ensure complete feature tables with a high peak abundance accuracy for each analysis performed.

## Supporting Information Available

Supplemental Methods; major steps of nontargeted feature detection and glossary; benefits of isotopologues in benchmarking nontargeted feature detection; quality metrics for mzRAPP BMs; impact of isotopologues on BM sizes and distributions of chromatographic FWHM and mz ranges; different variables extracted for all BM peaks; impact of the number of molecules used for BM generation; impact of MZmine 2 parameters on feature detection performance; and overview of used datasets and BMs (PDF). Visual definitions; information regarding generated BMs; and feature extraction results (XLSX). (ZIP)
