# Supplementary material for: Power of mzRAPP-Based Performance Assessments in MS1-Based Nontargeted Feature Detection
Source: Anal Chem. 2022 Jun 7;94(24):8588–95. doi: 10.1021/acs.analchem.1c05270 (PMC9218958; doi:10.1021/acs.analchem.1c05270)
Supplement: Supplementary file 1 — ac1c05270_si_001.zip [file ac1c05270_si_001.zip › Supplementary Information_revision_final.pdf]

Supporting Information

The power of mzRAPP-based performance assessments in MS1 based non-targeted feature detection

Yasin El Abiead[1,*], Maximilian Milford[1], Harald Schoeny[1], Mate Rusz[1,2], Reza M Salek[3], and Gunda Koellensperger[1,*]

1 Department of Analytical Chemistry, University of Vienna, Vienna 1090, Austria, 2 Department of Inorganic Chemistry, University of Vienna, Vienna 1090, Austria, and 3 International Agency for Research on Cancer, Section of Nutrition and Metabolism, Lyon 96008, France

Table of Contents:

Supplemental Methods & Information.

Figure S1. Major steps of non-targeted feature detection and glossary

Figure S2. Benefits of isotopologues in benchmarking non-targeted feature detection

Figure S3. Quality metrics for mzRAPP benchmarks

Figure S4. Impact of isotopologues on benchmark sizes and distributions of chromatographic FWHM and mz ranges

Figure S5. Different variables extracted for all benchmark peaks

Figure S6. Impact of number of molecules used for benchmark generation

Figure S7. Impact of MZmine 2 parameters on feature detection performance

Table S1. Overview of used datasets and benchmarks

Supplemental Methods

Sensitivity of NFD extraction parameters

Dataset (DS) 6, which consists of 9 replicate injections of the same sample, was processed via XCMS3 (version 3.14.1) using R 4.1.0, and MZmine 2 (version 2.53). For XCMS3 we applied the Centwave peak detection algorithm with prefilter = c(3, 100), ppm = 5, noise = 5, snthresh = 3, fitgauss = TRUE, integrate = 2 and mzdiff = -0.002. In the peakwidth parameter we set the minimum allowed peak width to 1. The maximum allowed peak width was increased from 6 to 30 s with steps of 2 s. All other parameters were at their default values. Alignment of peaks was performed via the PeakDensity algorithm with minFraction = 0.1, and binSize = 0.002. For the bandwidth (bw) parameter the values 6, 12 and 18 were tested. All other parameters were left at default. Filling of gaps was done via the fillChromPeaks algorithm, with all parameters left at their default values. For MZmine 2, mass detection was performed via Mass detector = Centroid and Noise level = 0. ADAP Chromatogram builder was used for chromatogram extraction with Min group size = 5, Group intensity threshold = 1000, Minimum highest intensity = 15000, and m/z tolerance set to 0.003 m/z or 5 ppm. Peak detection was performed via Local Minimum Search with a chromatographic threshold = 30%, Search minimum in RT range = 0.2, Minimum relative height = 5%, Minimum absolute height = 15000, Minimum ratio peak top/edge = 2, and Peak duration range starting from 0.05 min. The maximum allowed peak duration was screened using values from 0.2 to 2, with a step size of 0.2. Peak alignment was performed via Join aligner with m/z tolerance = 0.003 or 3 ppm, weight for m/z = 0.8, weight for RT = 0.8 and Retention time tolerance values 4, 10 and 15 s. For the extraction of performance metrics via mzRAPP, we used benchmark (BM) 6.

Coefficient of Variance investigation

DS 6 was processed via XCMS3 in the same manner as described for the parameter sensitivity study, but with different maximum allowed peak width and bw values. Specifically, maximum allowed peak width values were screened from 5 to 30 s with a step size of 1 s, and bw values were set to 0.5, or 2. For the extraction of performance metrics via mzRAPP, we used benchmark 6, but filtered it to contain only features with a peak in each sample and a Coefficient of Variance < 30%, as calculated via chromatographic peak areas.

Application of parameter optimization tools

Parameters for non-targeted feature detection in DS 6 were optimized via IPO (version 1.18.0), AutoTuner (version 1.6.0), MetaboanalystR 3.0, and SLAW (version 1.0.0). For IPO parameters were optimized once from default starting values of IPO and once with slightly adapted starting values. Specifically, min_peakwidth was set to 3-8, max_peakwidth to 30-50 ppm to 1-5, noise to 0-5000, bw to 3-20, mzwid to 0.005-0.02, and minfrac to 0.1. Optimized parameters were then applied via XCMS. For AutoTuner lag was set to 22, threshold to 3.1 and influence to 0.15 for initial processing. For ‘default’ application of AutoTuner we only set parameters optimized by AutoTuner in XCMS3 and left the rest at the default values from XCMS3. In a second attempt we manually set the binSize of the PeakDensity algorithm in XCMS3, which is not optimized by AutoTuner, to 0.005. For

optimization via MetaboanalystR 3.0 we set the starting parameters via their implemented platform selection to ‘UPLC-Q/E’, and set the polarity to ‘positive’, and the minFraction to 0.2. Non-targeted feature detection was then conducted within the MetaboanalystR 3.0 package. For SLAW we set the peak detection algorithm to ‘centwave’, and optimization = true. Non-targeted feature detection was then conducted within SLAW.

Assessment of NeatMS

NeatMS was run via Python 3.7. A model for NeatMS was generated from peaks generated from DS 1 via XCMS3. In total 164 ‘High Quality’, 57 ‘Low Quality’, and 1497 ‘Noise’ peaks have been labelled for training. We then trained the network via Transfer Learning, using default parameters as provided in the NeatMS tutorial for all parameters. Afterwards, NeatMS was applied to label the same 9 aligned XCM3 outputs generated for the unsupervised clustering investigation, described above. Using R, different filters were applied to the NeatMS output so each NFD feature was required to contain a minimum of 0, 1, 3, 5, 8, or 10 ‘High Quality’ peaks, while all other features were removed. For the extraction of NFD performance metrics via mzRAPP, BM 1 was filtered so it contained only features which contained a peak for each of the 10 samples.

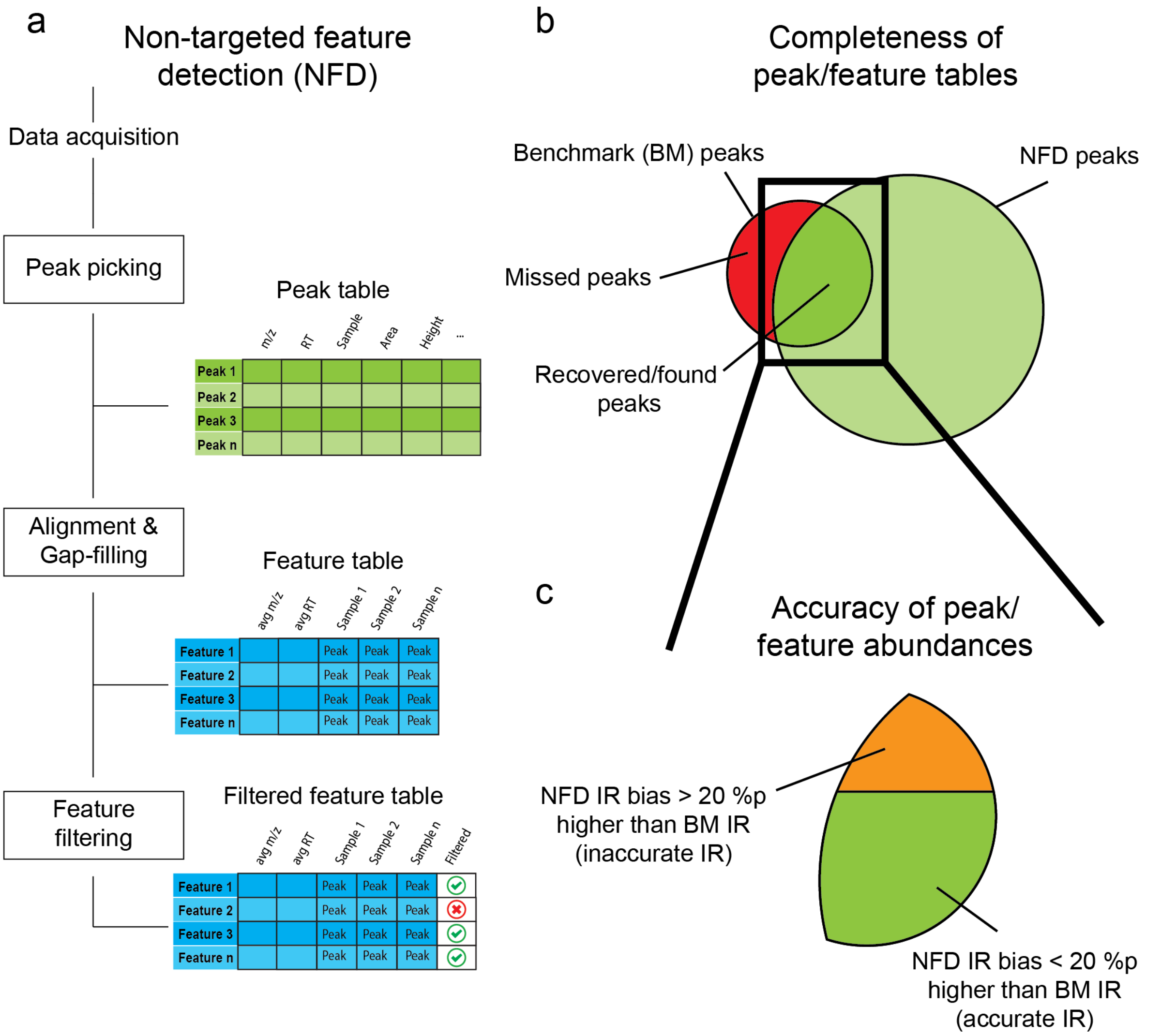

Figure S1. Major steps of non-targeted feature detection (NFD) and glossary: (a) Chromatographic peaks with an associated retention time and mz value are referred to as peaks. Multiple peaks aligned across several samples with an associated average mz and retention time value are referred to as features. Peak-picking, alignment and gap-filling can be performed via tools such as XCMS, while feature filtering can be performed by a variety of methods such as via Coefficient of Variance or deep-learning based peak classification (discussed in a later chapter). (b) The completeness of NFD results is determined by assessing the overlap between NFD results and benchmark (BM). (c) The accuracy of peak abundances is determined by assessing the proportion of BM isotopologue ratio (IR) biases increased by > 20 percentage points (%p) in the NFD results.

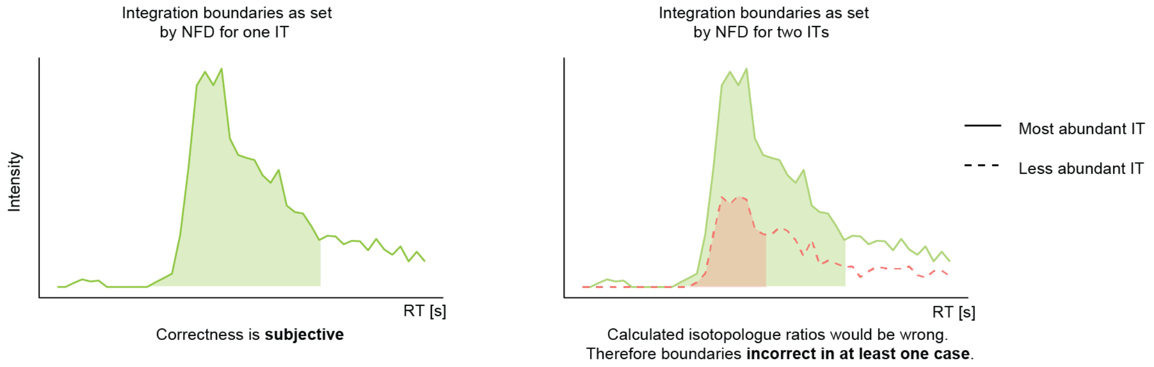

Figure S2. Considering isotopologues (ITs) for non-targeted feature detection (NFD) reliability assessment brings several key advantages. (a) By relying on IT ratios rather than exact benchmark (BM) peak areas, the evaluation of set integration boundaries becomes less subjective.

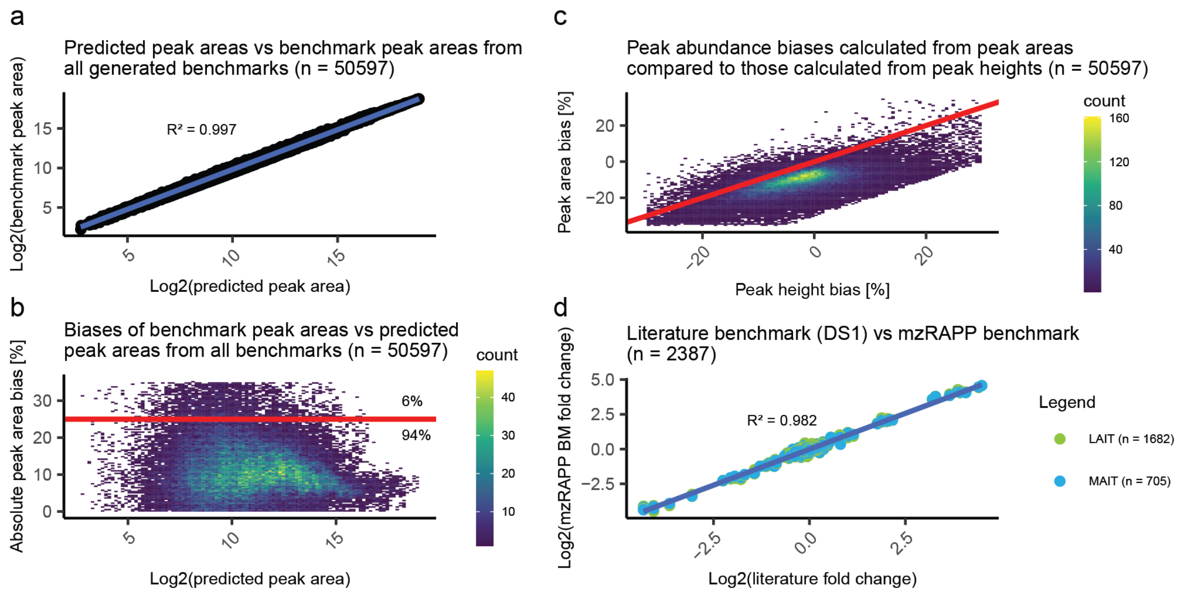

Figure S3. Plots characterizing benchmarks (BM) generated via mzRAPP. The peak area/height of low abundant isotopologues (LAITs) can be predicted from the peak area/height of the respective most abundant isotopologue (MAIT) when the molecular composition is known. This is done by multiplying the observed area or height of the MAIT with the relative factor for a given LAIT (as known from the molecular composition). (a) The plot demonstrates that this linear relationship is given for all peaks in mzRAPP generated BMs. (b) The plot shows how the relative bias of this prediction is below 25% for 94% of all BM peaks. (c) Small differences in biases calculated from peak heights to those from peak areas form an additional line of evidence for high-quality peak abundances. (d) Fold changes between two sample groups published for dataset 1 were reproduced by mzRAPP not only for the reported MAIT, but also for LAITs underlining mzRAPPs ability to produce peaks with reliable abundances.

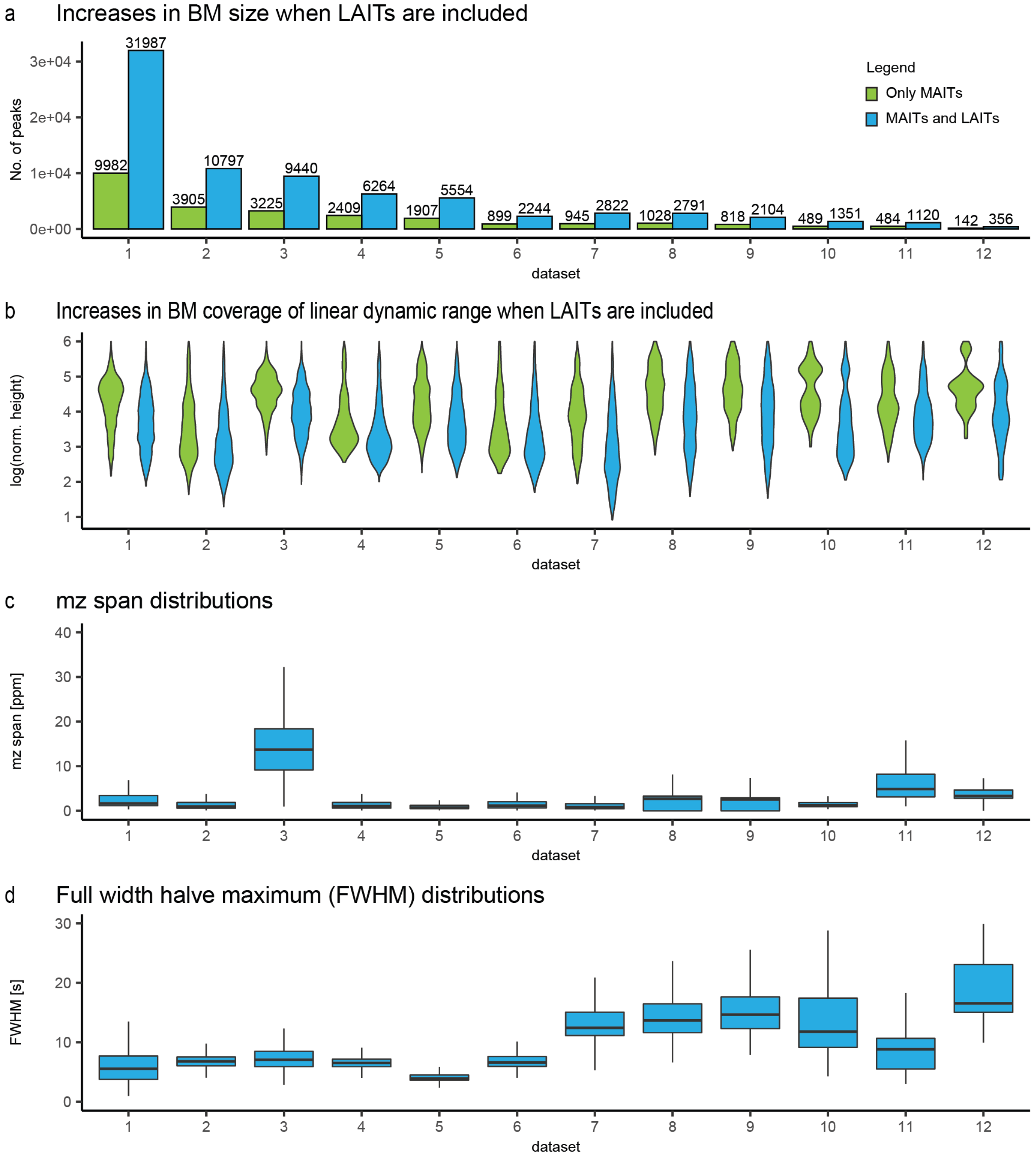

Figure S4. Eleven different benchmarks (BM) from different data sets (1-12, see supplement) have been produced using mzRAPP. (a) The bar plot shows the increased number of peaks achieved by adding peaks from all detectable isotopologues (blue) compared to only considering the most abundant isotopologue (MAIT). (b) The addition of lower abundant isotopologue (LAIT) peaks increased the benchmark coverage, as can be seen in the violin plots visualizing the linear dynamic range. In some cases, the covered linear dynamic range increased about two orders of magnitude. (c & d) Finally, the distribution of full width at half maximum (FWHM) and mz range is shown for all BM peaks.

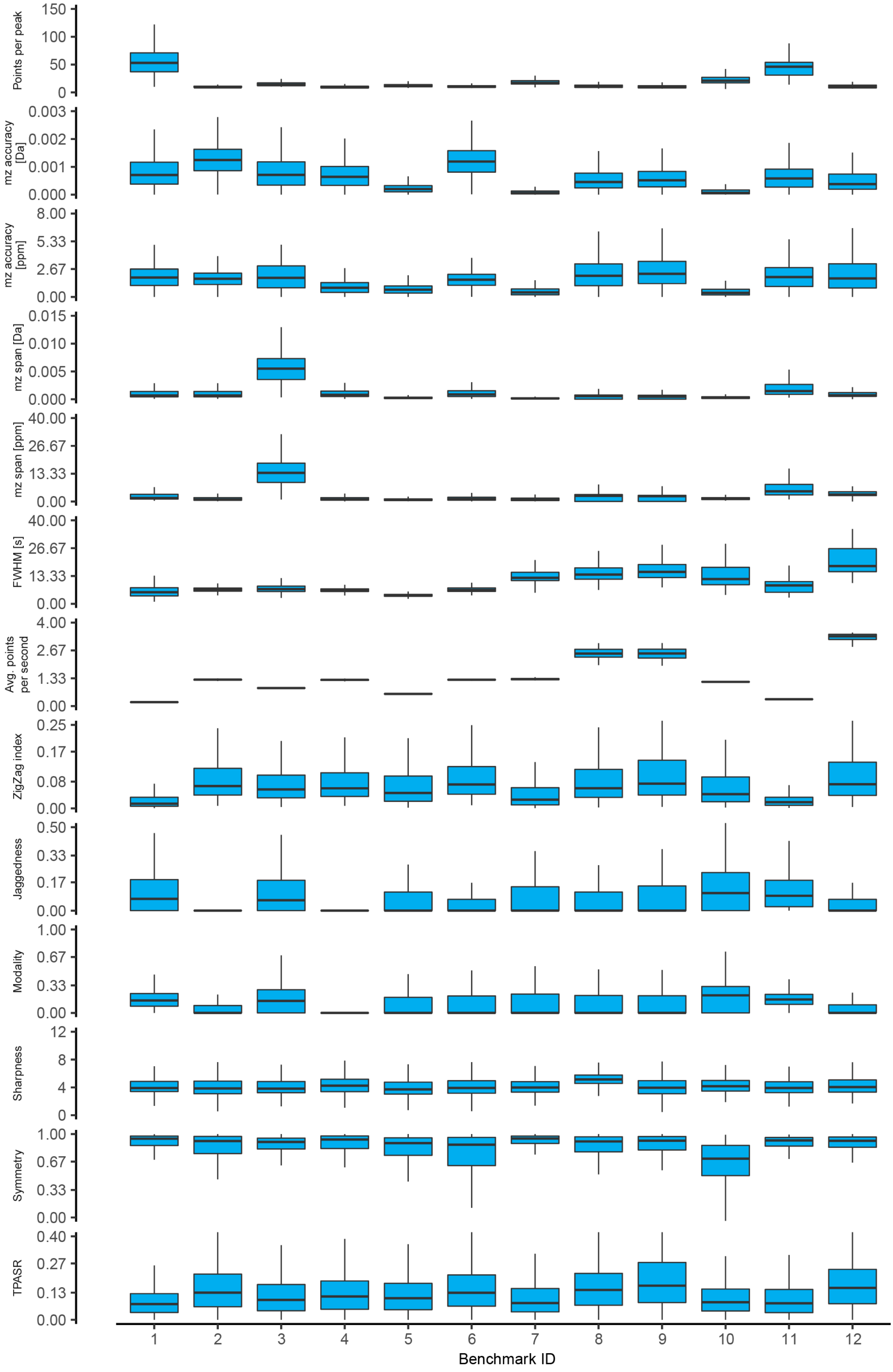

Figure S5. Distribution of peak variables for all generated benchmarks (outliers not shown).

Impact of BM molecule number on mzRAPP metrics

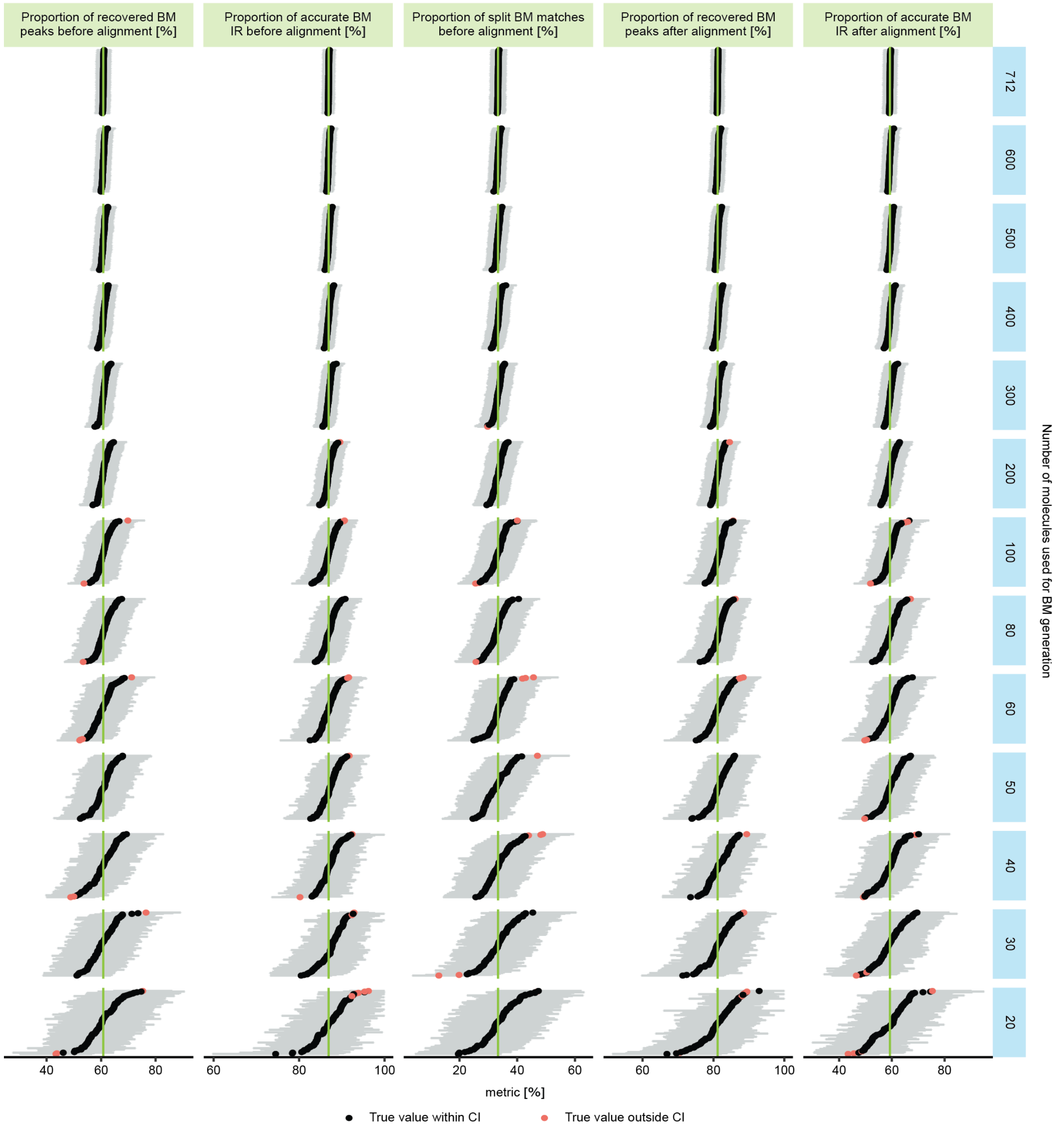

Figure S6. A benchmark was generated for dataset 1 considering 712 molecules and used for NFD performance assessment. Stepwise reduction of molecules used for BM generation (molecules were sampled at random from all 712 molecules (n = 100) for molecule numbers ranging from 20 to 712) largely affected the size of confidence intervals (CI; as estimated by mzRAPP via bootstrapping with R = 1000 and confidence level = 0.99), while true metrics, as estimated from the original BM (green lines) stayed largely within the reported CI of respective NFD assessments. (CI values above 100 were reduced to 100)

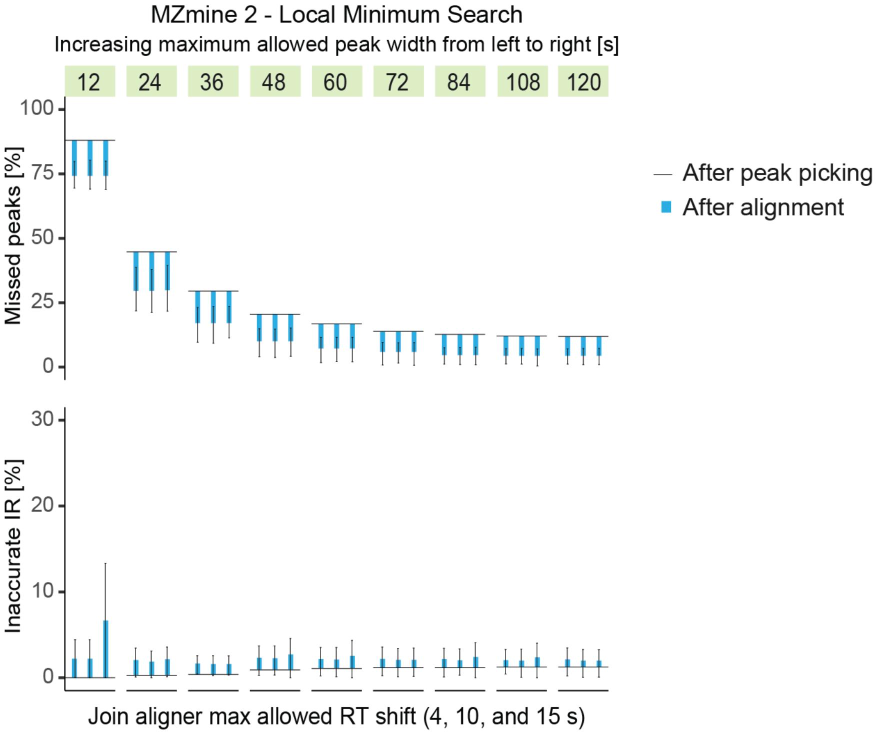

Figure S7. Multiple non-targeted feature detection experiments with parameter sweeps for the peak-picking and the alignment step were performed on the same dataset via MZmine 2. The peak picking parameter referring to the maximum peak width allowed (MPW) was incrementally increased for both tools, with three retention time tolerances tested for each peak picking step. For each parameter combination proportions of missed peaks and inaccurate isotopologue ratios are shown for before and after the alignment step.

Table S1. Overview of used datasets and generated benchmarks

Dataset/Benchmark ID	Chromatography	Mass spectrometer	Polarity	Samples	Molecules	Peaks	Features	Reference
1	ZORBAX Eclipse C18 (RP)	Q Exactive HF	pos	10	712	31987	3513	[13]
2	ACQUITY HSS T3 (RP)	Q Exactive HF	pos	59	104	10797	388	MTBLS 1876 [18]
3	ZORBAX Eclipse C18 (RP)	Sciex TripleTOF6600	pos	8	451	9440	1725	[13]
4	ACQUITY HSS T3 (RP)	Q Exactive HF	neg	60	80	6264	242	MTBLS 1876 [18]
5	Kinetex EVO C18 (RP)	Q Exactive Plus	pos	49	195	5554	919	MTBLS 1455 [15]
6	ACQUITY HSS T3 (RP)	Q Exactive HF	pos	9	119	2244	366	MTBLS 1876 [18]
7	SeQuant ZIC pHILIC	Q Exactive Plus	pos	26	77	2822	338	MTBLS 706 [19]
8	SeQuant ZIC pHILIC	LTQ Orbitrap	pos	30	42	2791	154	MTBLS 265 [20]
9	SeQuant ZIC pHILIC	LTQ Orbitrap	pos	30	36	2104	126	MTBLS 267 [20]
10	SeQuant ZIC pHILIC	Q Exactive HF	neg	24	26	1351	96	MTBLS 2672 [21]
11	ACQUITY HSS T3 (RP)	Sciex X500R QTOF	pos	15	44	1120	126	MTBLS 1785 [11]
12	SeQuant ZIC pHILIC	LTQ Orbitrap	neg	30	10	356	28	MTBLS 265 [20]

Reference IDs refer to the reference section in the main publication. A detailed list of the raw files in each benchmark is included in Table S3.
